# On a Neglected Symptom in Breast-Cancer

**Published:** 1881-01

**Authors:** Herbert L. Snow


### Selections.

### ON A NEGLECTED SYMPTOM IN BREAST-CANCER.

BY HERBERT L. SNOW, M. D.

I DESIRE to call attention to a symptom which very commonly occurs in the course of breast-cancer, and on which, I think, sufficient stress has not hitherto been laid. Beyond a general allusion to the implication of the osseous system in the later stages of cancer, I have not met with any description of this symptom in surgical works, but from its frequency and obviousness I can hardly presume that it has not been noticed in numerous instances, I doubt, however, whether the earliness of its appearance has been remarked, and whether its importance as a prognostic sign has been duly realized.

I refer to a thickening of the humerus on the side correspond- to the diseased gland, accompanied by tenderness on pressure· This condition obtains mainly over the trochanters and the upper third of the bone. On firm pressure the patient complains of tenderness, which tenderness extends for a variable distance down the shaft, beyond the part where thickening is apparent. The tenderness and thickening rarely interfere with the movements of the arm, and are never noticed by the patient before examination; they are only detected by digital pressure and comparison with the humerus on opposite side. Occasionally (but not often) there is also some thickening of the clavicle. The condition never advances to any very marked hypertrophy.

These symptoms are found in the majority of cases of ordinary breast-scirrhus comparatively early in the course of the disease, and simultaneously with commencing enlargement of the axillary glands. I have lately operated on a case of four months (stated) duration, in which two axillary glands were about the size of a horse-bean; the others (all of which were removed, as far as possible), and being not manifestly affected, yet there is already some thickening of the humerus, with tenderness extending down half the shaft. The bony thickening thus appears, as a rule, long before œdema of the arm. When that has supervened, the condition, of course, is completely masked, and when it takes place, as in a few cases comparatively early, may not be noticed at all; though I believe that it is an invariable concomitant of the disease sooner or later. I may add that in a few cases the tenderness on pressure is more obvious than the bony enlargement.

I have not yet had an opportunity of examining microscopically one of these cases before it has run its usual course, and the brawny œdema of the arm has set in. After death with such a condition I have found the medulla of the affected bone red in color, and completely composed of nearly spherical cells containing very large nuclei, without any fat cells on the one hand, and on the other without alveolar structure. There was no obvious hypertrophy of the bone after removal.

In the later stages of cancer, severe pains in the thighs (so-called sciatica), pelvis, and lumbar spine have long been noticed as a proof of advanced systemic implication; there is great fragility of all the bones, and often a pseudo-paralysis of the lower limbs. This condition, doubtless, is but a later stage of the one I have described; although it becomes noticeable only in a certain proportion of cases, being usually masked by other symptoms. The influence of secondary carcinoma on the osseous system has not been worked out; but in considering it, we naturally remember the frequency with which we find primary cancer of bone attended by secondary deposits in distant parts of the skeleton, yet with little or no affection of the other tissue. I do not pretend to explain how (in secondary cancer) the first contamination takes place—probably through some lymphatic channels hitherto undescribed. But I think the facts tend to indicate that the medulla of bones is a specially favorable nidus for the development of malignant disease; that when once cancer germs reach this they speedily multiply in the soft and vascular tissue; and that, not unfrequently, all the long bones become filled with cancerous material, whose presence is not always manifested by symptoms. Bearing in mind the reported developments of red blood-corpuscles in the medulla, it would be interesting to speculate on the influence which such a condition would have upon nutrition generally, and the familiar cancerous cachexia.

I am disposed to regard the "thickening" I have referred to as due to a low form of periostitis, consequent upon deposit of cancer germs in the medulla. I look upon it as only apparent, and do not think there is any real hypertrophy of the osseous tissue.

In all the cases I have noticed there has been a recurrence of the disease within a few weeks or months. The appearance indicates that the disease has extended beyond merely local treatment, and that a renewal of its more obvious manifestations at no distant date is a certainty. I do not consider the condition an absolute bar to operations, but it is undoubtedly one which ought to be previously taken into consideration, and, whenever present, operative measures must be described to the patient only in the light of a palliative. It is a symptom of grave prognostic importance, and my only excuse for offering these somewhat crude remarks is my wish to direct more general attention to a practical point hitherto little regarded.

